# Brain plasticity following MI-BCI training combined with tDCS in a randomized trial in chronic subcortical stroke subjects: a preliminary study

**DOI:** 10.1038/s41598-017-08928-5

**Published:** 2017-08-23

**Authors:** Xin Hong, Zhong Kang Lu, Irvin Teh, Fatima Ali Nasrallah, Wei Peng Teo, Kai Keng Ang, Kok Soon Phua, Cuntai Guan, Effie Chew, Kai-Hsiang Chuang

**Affiliations:** 1 Singapore Bioimaging Consortium, Agency for Science Technology and Research, 11 Biopolis Way, #02-02 Helios, Singapore, 138667 Singapore; 2 Institute for Infocomm Research, Agency for Science Technology and Research, 1 Fusionopolis Way, #21-01 Connexis (South Tower), Singapore, 138632 Singapore; 3 Clinical Imaging Research Center, Agency for Science Technology and Research, Centre for Translational Medicine (MD6), 14 Medical Drive, #B1-01, Singapore, 117599 Singapore; 4 Division of Neurology, National University Hospital System, 5 Lower Kent Ridge Road, Singapore, 119074 Singapore; 5 Yong Loo Lin School of Medicine, National University of Singapore, NUHS Tower Block, Level 11, 1E Kent Ridge Road, Singapore, 119228 Singapore; 6 Queensland Brain Institute and Centre for Advanced Imaging, The University of Queensland, Brisbane, Queensland 4072 Australia; 7 Present Address: Wellcome Trust Centre for Human Genetics, Roosevelt Drive, Oxford, OX3 7BN UK; 8 Present Address: Queensland Brain Institute, The University of Queensland, Brisbane, Queensland 4072 Australia; 9 Institute for Physical Activity and Nutrition (IPAN), Present Address: School of Exercise and Nutrition Sciences, Deakin University, 221 Burwood Highway, Burwood, VIC 3125 Australia; 10 School of Computer Science and Engineering, Nanynag Technological University, 50 Nanyang Avenue, Singapore, 639798 Singapore

## Abstract

Brain-computer interface-assisted motor imagery (MI-BCI) or transcranial direct current stimulation (tDCS) has been used in stroke rehabilitation, though their combinatory effect is unknown. We investigated brain plasticity following a combined MI-BCI and tDCS intervention in chronic subcortical stroke patients with unilateral upper limb disability. Nineteen patients were randomized into tDCS and sham-tDCS groups. Diffusion and perfusion MRI, and transcranial magnetic stimulation were used to study structural connectivity, cerebral blood flow (CBF), and corticospinal excitability, respectively, before and 4 weeks after the 2-week intervention. After quality control, thirteen subjects were included in the CBF analysis. Eleven healthy controls underwent 2 sessions of MRI for reproducibility study. Whereas motor performance showed comparable improvement, long-lasting neuroplasticity can only be detected in the tDCS group, where white matter integrity in the ipsilesional corticospinal tract and bilateral corpus callosum was increased but sensorimotor CBF was decreased, particularly in the ipsilesional side. CBF change in the bilateral parietal cortices also correlated with motor function improvement, consistent with the increased white matter integrity in the corpus callosum connecting these regions, suggesting an involvement of interhemispheric interaction. The preliminary results indicate that tDCS may facilitate neuroplasticity and suggest the potential for refining rehabilitation strategies for stroke patients.

## Introduction

Repetitive, task-specific motor training is one of the key components of post-stroke rehabilitation; however, active voluntary movement is challenging or even impossible for patients with severe motor impairment. By combining robot-assisted motor imagery and brain-computer interface (MI-BCI), we have developed a system to automatically drive the movement of the stroke-affected limb of a patient with a robotic arm via motor imagery of the arm which can be detected from electroencephalographic (EEG) recordings^1^. By providing multisensory feedback and facilitating integration with motor learning, MI-BCI has been demonstrated to be a promising tool for improving functional recovery in stroke patients^1–7^.

Another emerging technique for facilitating the functional recovery after stroke is transcranial direct current stimulation (tDCS). TDCS is a non-invasive brain stimulation technique that delivers weak direct current through two saline-soaked electrodes (anode and cathode) over specific areas of the scalp^8^. Studies have shown that anodal tDCS is able to induce an increase in corticospinal excitability, as measured by transcranial magnetic stimulation (TMS), while cathodal tDCS exerts a suppressive effect^9, 10^. More importantly, repetitive tDCS sessions may induce behavior changes that last for weeks^11, 12^. The long-lasting after effect of tDCS is thought to be associated with the long term potentiation that underlies the learning process; therefore, tDCS holds the potential for improving motor function recovery through modulation of brain plasticity. However, the exact mechanism is still elusive^9, 10^. Studies have shown that anodal tDCS on ipsilesional primary motor cortex (M1) or cathodal stimulation on the contralesional M1 may improve the motor recovery^13, 14^. Furthermore, a few studies suggested that anodal tDCS may enhance the detection accuracy of motor imagery by EEG^15–18^, which is a major limitation of MI-BCI. With the converging evidence, we investigated whether combining tDCS and MI-BCI may further improve neuroplasticity and functional recovery in stroke survivors with motor impairments.

To understand the neuroplasticity induced by the MI-BCI and tDCS, we applied neuroimaging to track the structural and functional recovery following the rehabilitation. Diffusion Tensor Imaging (DTI), by measuring the random movements of water molecules (i.e., diffusion) in tissues, is sensitive to the microscopic structural, compositional, and organizational changes of the white matter (WM) in the brain^19^. There are several commonly used DTI metrics, including the parallel diffusivity (Dp), radial diffusivity (Dr), and fractional anisotropy (FA). Dp is related to the axonal integrity, while Dr is associated with factors such as membrane permeability, myelin thickness, and axonal density. FA is usually thought to reflect the overall integrity of the axonal fibers. These DTI metrics could provide useful information in stroke patients to determine the extent of the WM lesion^20^, the relationship with functional recovery^21, 22^, and process of intervention/recovery^23^. Cerebral blood flow (CBF) is an indicator of the cerebrovascular function and neural activity due to its tight coupling with the cerebral metabolism. Arterial spin labeling (ASL) is a non-invasive MRI technique for quantitative measurement of CBF without the usage of exogenous contrast agent. While numerous studies have investigated the disruption of CBF during acute or subacute phase of stroke, limited studies have looked at CBF in the chronic stage^24–26^. Two case reports have related the CBF deficits with functional impairments^25, 27^. One study reported interhemispheric rebalance of CBF during the functional reorganization and recovery after unilateral stroke^28^.

In this study, we applied DTI, ASL and TMS to understand the changes in gray and white matter and excitability of the corticospinal pathway before, immediately after 2 weeks of anodal tDCS and MI-BCI and 4 weeks post-intervention in moderate-to-severe stroke patients with upper limb disability. We hypothesized that the combination of tDCS with MI-BCI training will induce greater and longer-lasting neuroplasticity in the white matter tracts, gray matter function, and excitability of the corticospinal pathway compared to MI-BCI alone. Our results show that adding tDCS can induce long-lasting changes in cortical excitability and CBF, as well as enhance the integrity in white matter connecting the bilateral sensorimotor cortices.

## Materials and Methods

### Subjects

**Figure 1:**
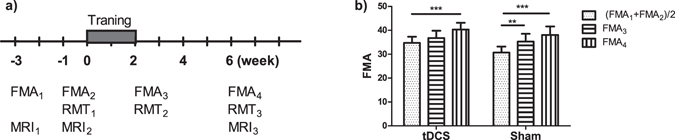
Study design and behavior outcome. (**a**) timeline of the training, clinical assessment of FMA score, resting motor threshold (RMT), and MRI. (**b**) Both tDCS and sham groups produce increase in the FMA after training. **p < 0.01; ***p < 0.001. Error bar represents SEM.

The study was in compliance with the Code of Ethics of the World Medical Association, and approved by the Domain Specific Review Board (DSRB) of the National Healthcare Group, Singapore (Clinical Trial Registration Unique Identifier: NCT01897025, date of registration: July 8, 2013; https://clinicaltrials.gov/ct2/show/NCT01897025). Sample size was determined by our preliminary results and other studies that used similar endpoints. Forty-two subjects were assessed for eligibility between June 2011 to January 2014. Eight of them declined to participate, 5 were excluded because they did not meet the inclusion criteria, and another 10 were excluded because they did not meet the BCI performance criteria. The remaining 19 subjects (54.1 ± 10.8 years old, 5 female) completed the training and MRI scans. Written informed consent, in which the nature of the experimental procedures was explained, was obtained from all participants. The subjects had their first ever subcortical stroke more than 9 months prior to study enrollment that led to unilateral moderate to severe impairment of upper extremity as scored 11–45 in Fugl-Meyer assessment (FMA). Subjects exclusion criteria include epilepsy, neglect, cognitive impairment, other neurological or psychiatric diseases, severe arm pain, spasticity score >2 on the Modified Ashworth Scale in the shoulder or elbow, contraindications to TMS or tDCS (cranial implants, ventricular shunts, pacemakers, intrathecal pumps), grip strength <10 kg as measured by a dynamometer or participation in other interventions or trials targeting stroke motor recovery. Among the 13 subjects included in CBF analysis (see below), 5 of them had no significant arterial stenosis, and 1 had mild stenosis in the ipsilesional MCA. There was no stenosis diagnosis information available for the remaining 7 subjects, but they did no show significantly reduced baseline CBF, post-training CBF changes, or bilateral CBF differences compared to other patients (See Supplementary Figure S1). The subjects went through motor function and corticospinal excitability assessments, study interventions and MRI following the timeline shown in Fig. 1a. In addition, 11 healthy subjects (57.2 ± 5.0 years old, 5 female) were recruited as controls with written informed consent. Only two MRI scans (3 weeks apart) were conducted in the healthy controls to evaluate reproducibility. All screening and study procedures were performed at the National University Hospital, Singapore, except that MRI was performed at the Clinical Imaging Research Center, Singapore.

### Motor function assessment

For stroke subjects, motor function of the affected arm was evaluated by the upper extremity component of the Fugl-Meyer assessment at the initial screening, 1 week prior to, immediately after and 4 weeks after the training (Fig. 1a). The mean of the first 2 measurements was taken as the pre-training score, and the last measurement as the post-training score. Linear mixed effects analysis of the FMA with Dunnett’s post test was performed using R (v3.3.0, https://www.r-project.org/).

### MI-BCI and tDCS Intervention

Stroke subjects were grouped according to their pre-training FMA scores (11–28 and 29–45) and then randomly assigned using a computer-generated random sequence into tDCS or sham group with matching FMA score between the groups. Each subject underwent ten 40-minute sessions of MI-BCI training over 2 weeks, each session preceded with either 20 minutes of tDCS or sham-tDCS (current ramped up and down to give subjects the sensation of the stimulation), applied at 1 mA through a pair of saline-soaked surface sponge electrodes (35 cm^2^), as per published protocol^29^. The anode was placed over the ipsilesional M1 while the cathode over the contralesional M1, according to the initial exploration using TMS which identified the hotspot for activating the muscle of the hand and with reference to the International 10–20 Electrode Placement System for EEG electrode placement. The MI-BCI training involved mental imagery of a reaching task. Motor intention was detected using EEG, which triggered the movement of the stroke-affected arm using the Inmotion^2^ MIT-Manus robot (Interactive Motion Technologies, MA, USA)^30^. As EEG signals were continuously recorded during the MI-BCI training and tDCS may interfere the detection accuracy of EEG, the tDCS was applied prior to the MI-BCI training. The patient and the assessors were blinded to the tDCS condition.

### Single and paired-pulse TMS

For stroke subjects, resting motor threshold (RMT) of both arms was measured by single-pulse TMS at 3 time points: 1 week prior to, immediately after and 4 weeks after the intervention. RMT is defined as the percentage of the maximum stimulator output required to elicit motor evoked potential (MEP) with 50–100 µV peak-to-peak amplitude in at least 4 out of 8 trials using Bistim 200^2^ (Magstim Co., UK). A lower stimulator output to elicit RMT indicates a higher degree of corticospinal excitability. All Subjects were seated upright on a chair and was instructed to keep still and relaxed during the TMS measurement. Subjects were also instructed to place both the left and right arms comfortably on a pillow on their laps and to remain as relaxed as possible. A 70 mm figure-of-eight coil was placed on the scalp at a 45° orientation and the optimal scalp position for activating the abductor pollicus brevis (APB). The optimal location of the APB on the M1 was determined from initial exploration over a 10-mm grid marked on a cap. MEP amplitudes were recorded from APB via surface electrodes in a belly-tendon arrangement, using Medelec Synergy EMG system (VIASYS Healthcare, UK). Short intra-cortical inhibition (SICI) and intracortical facilitation (ICF) were measured using paired pulse TMS with an initial conditioning stimulus of 80% of RMT and a test stimulus of 120% of RMT separated by an inter-stimulus intervals (ISIs) of 2ms to induce SICI, while an ISI of 10 and 15ms were used to elicit ICF. Due to the high stimulator output, fewer trials was used in order to reduce the level of discomfort. No motor function and cortical excitability assessment, nor MI-BCI tDCS was performed in healthy subjects. Linear mixed effects analysis of the RMT was performed using *R*.

### MRI

MRI data were collected using a 3T scanner (TIM Trio, Siemens, Germany) with a 32 channel head array coil at 3 time points: at screening, 1 week prior to, and 4 weeks after the training. Perfusion images were acquired using pseudo-continuous arterial spin labeling (pCASL) with the labeling duration = 1500 ms, post-labeling delay = 1500ms, and gradient-echo EPI of TR = 4000 ms, TE = 9.1 ms, GRAPPA factor = 3, voxel = 3 × 3 × 5 mm^3^, and 40 label-control pairs. DTI data were acquired with spin-echo EPI with 61 diffusion sensitizing directions, *b* = 1000 s/mm^2^, TR = 8000 ms, TE = 87 ms, GRAPPA factor = 2, and voxel size = 2.3 mm isotropic. T1-weighted images were acquired with a magnetization prepared rapid gradient-echo (MPRAGE) sequence in the sagittal view with TI = 900 ms, TR = 1900 ms, TE = 2.5 ms, and voxel size = 1mm isotropic. T2-weighted images were acquired with fluid-attenuated inversion recovery (FLAIR) sequence in the coronal view with TR = 9320 ms, TE = 82 ms, and voxel size = 0.9 × 0.9 × 3 mm^3^.

### Data Analysis

**Figure 2:**
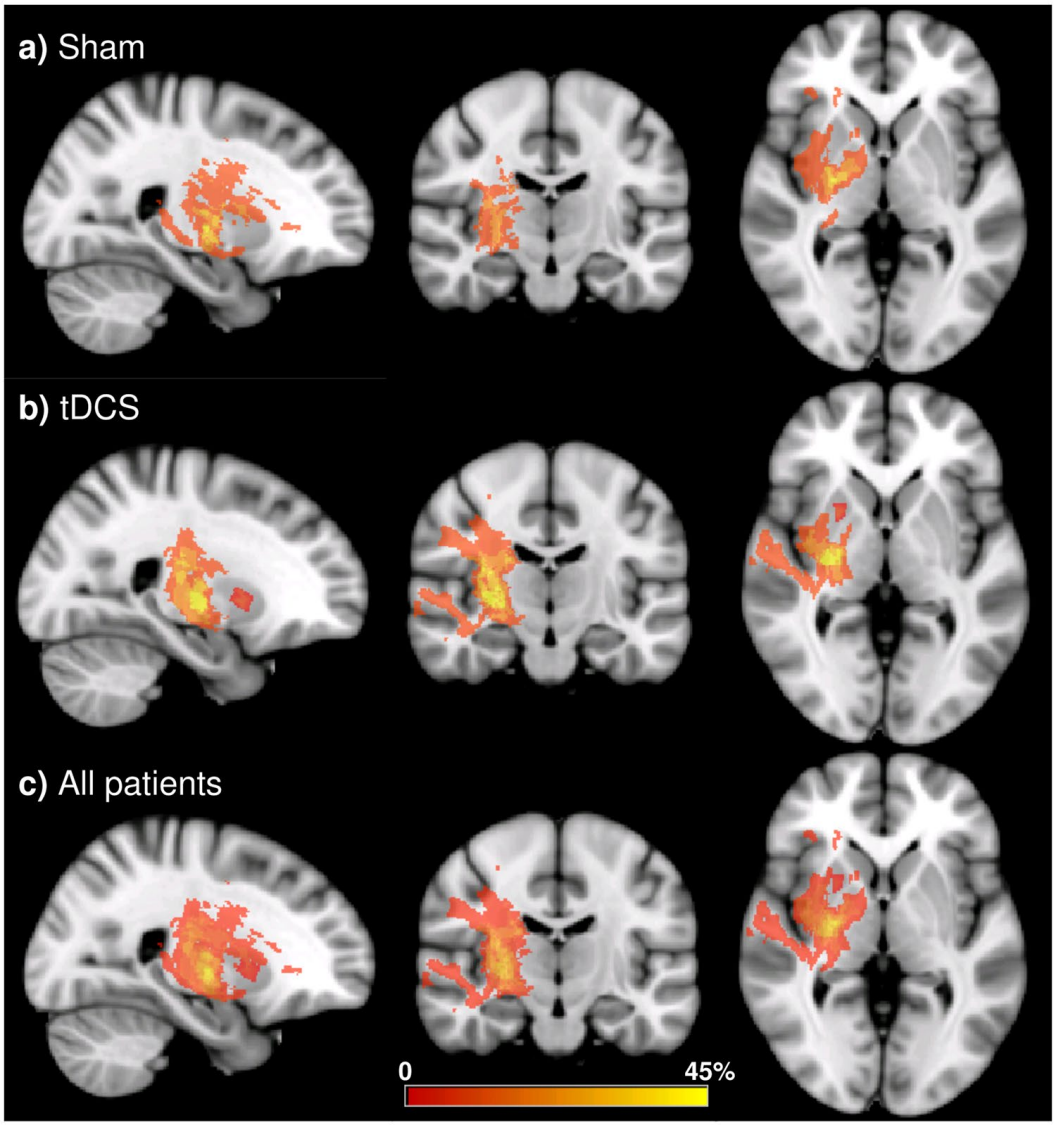
Incidence maps of lesion. The overlapping proportion of lesions from (**a**) the sham group, (**b**) the tDCS group, and (**c**) all patients combined, overlaid on the FMRIB58_FA template. Red and yellow colors denote low and high frequency of occurrence.

MRI processing and analysis was performed using FSL (ver5.0.7, http://fsl.fmrib.ox.ac.uk/)^31^ and in house MATLAB (The MathWorks, USA) codes. A binary lesion mask was manually delineated based on the T_2_-weighted FLAIR image to improve nonlinear image registration of all the data into the MNI152 template space by excluding the lesion areas from warp estimation (using the–inmask option in the FSL fnirt command). For subjects with lesion on the left hemisphere, images were flipped along the midline so that the lesion appeared on the right hemisphere for all subjects (Fig. 2).

**Figure 3:**
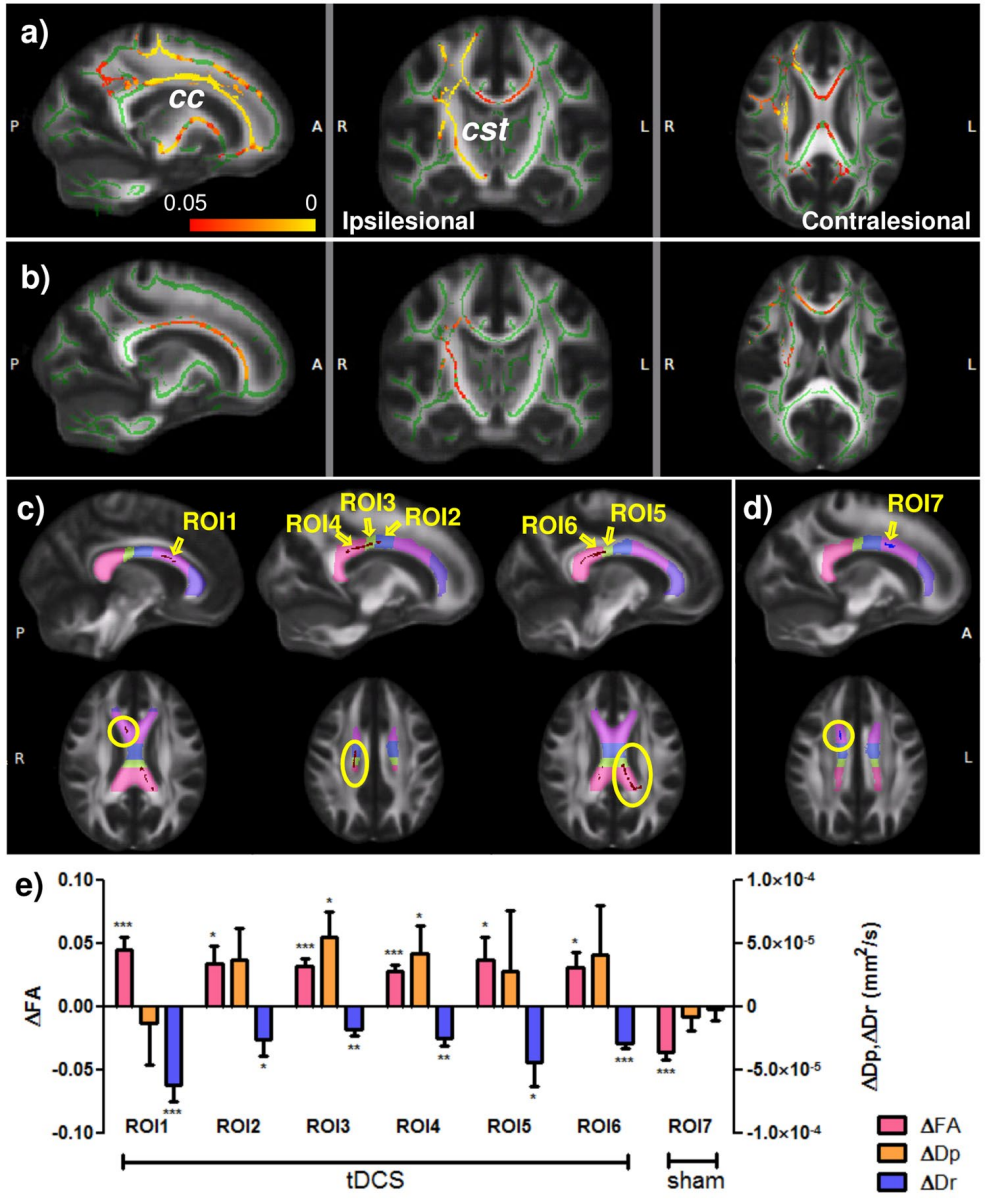
DTI results. (**a**) FA at baseline in all patients is significantly lower (highlighted in red-yellow) compared to healthy control (p < 0.05 FWE corrected). (**b**) tDCS group has larger FA increase in the *cc* and *cst* than the sham group (p < 0.05 FWE corrected). Lesion is on the Right side. The white matter skeleton (in green) is superimposed on the FMRIB58_FA template. *cc*: corpus callosum; *cst*: corticospinal tract. (**c**) and (**d**) White matter diffusivity change in the corpus callosum. (**c**) ROIs for the tDCS group in the 2^nd^ (ROI1), 3^rd^ (ROI2), 4^th^ (ROI3), and 5^th^ (ROI4) section of the ipsilesional *cc*, and in the 4^th^ (ROI5) and 5^th^ (ROI6) section of the contralesional *cc*. (**d**) ROI for the sham group in the 2^nd^ section of the ipsilesional *cc* (ROI7). Segmentation of the *cc* is denoted by colors as such: connecting prefrontal cortex (purple); premotor and supplementary motor cortices (magenta); motor cortex (blue); sensory cortex (green); and parietal, temporal, and occipital cortices (pink). (**e**) The tDCS group shows significant increase of FA due to changes in Dp and/or Dr in the ROIs. No Dr or Dp change was found in the region with decreased FA in the sham group. *p < 0.05; **p < 0.01; ***p < 0.001. Error bar represents SEM.

For DTI, after motion and eddy current artifact corrections by affine image registration, the tensor model was fitted to obtain FA, Dr., and Dp. Voxel-wise tract-based spatial statistics^32^ was then carried out on these metrics. Permutation tests were performed with threshold-free cluster enhancement and family-wise error rate controlled to a significance level of p < 0.05. Furthermore, the medial part of the corpus callosum (*cc*) was segmented into 5 functional sections from anterior to posterior: 1st connecting the prefrontal cortices, 2nd connecting the premotor and supplementary motor cortices, 3rd connecting the motor cortices, 4th connecting the sensory cortices, and 5th connecting parietal, temporal, and occipital cortices (Fig. 3c,d)^33^. In order to check the possible causes leading to FA changes, clusters showing significant FA changes in the *cc* were divided into regions of interest (ROIs) according to the segmentation atlas. The changes of the mean FA, mean Dp, and mean Dr over these ROIs were calculated and correlated with behavior scores.

Motion correction of the pCASL images was performed through a rigid registration with the 1st volume of the series as the reference. The brain of the mean image was extracted to create a brain mask, which was then applied to the entire 4D series. After a 2nd round of motion correction on this brain extracted series, CBF was quantified^34^. In order to minimize the artifacts by imperfect labeling and residual motions not corrected, a threshold of the whole brain mean CBF plus 3 standard deviations was applied to the CBF map, such that those hyperintensities exceeding the threshold were replaced by the mean of the neighboring voxels. The CBF map was then flipped (if lesion on the left hemisphere) and transformed to the MNI152 space by a combination of an affine registration from the flipped mean control image to the MPRAGE image and a nonlinear registration from the MPRAGE image to the MNI152 template. A study specific gray matter (GM) mask was generated by combining a binary GM mask based on the Harvard-Oxford probabilistic cortical structural atlas thresholded at 25%^35^ and a brain coverage map covering more than 90% of the subjects. The GM mask was applied to the registered CBF images after 2D Gaussian smoothing with FWHM of 6mm. Voxel wise group comparisons on the CBF maps were carried out with multiple comparison correction based on Gaussian random field theory, with a significance threshold of p < 0.05 at both voxel and cluster levels. The CBF maps were further correlated with the FMA scores. In addition, ROI based analysis was performed in the primary motor cortex and primary somatosensory (S1) cortex as defined by the Harvard-Oxford cortical structural atlas. For each ROI, relative CBF change was calculated as the difference between the post and pre-training CBF normalized by the latter. A CBF asymmetry ratio was calculated as the contralesional mean CBF over the sum of both sides.

Although all patients had their lesion core in the subcortical area, one stroke subject was removed due to excessive lesion extending to cortical areas, resulting in 9 stroke subjects in each group of the DTI data. For the CBF analysis, additional 5 subjects were excluded due to excessive head motions or unsuccessful labeling, resulting in 7 subjects in the tDCS and 6 in the sham group. Since no significant difference between the two pre-training scans (data not shown) in the FA or CBF map, the mean of the two pre-training data was averaged as the baseline.

### Data availability

The datasets generated during and/or analyzed during the current study are available from the corresponding author upon reasonable request.

## Results

### Clinical outcome

The demographic and clinical data are listed in Table 1. There was no difference between the tDCS and sham groups in terms of age (52.8 ± 12.3 vs. 56.4 ± 9.6 years, p = 0.49), post-stroke time (33.9 ± 24.6 vs. 33.3 ± 15.1 months, p = 0.95), or baseline FMA (34.7 ± 7.8 vs. 30.7 ± 7.4, p = 0.28). Linear mixed effects analysis on the FMA indicated significant time effect (p < 0.0001), but insignificant effect of brain stimulation (p = 0.50). In other words, subjects from both groups showed improved motor function, with no group difference either immediately or 4 weeks post training (Fig. 1b).

**Table 1:** Demographic and clinical data.

tDCS/Sham	Subject ID	Age (years)	Gender	Post stroke time (months)	Affected arm	Handed-ness					Resting motor thresholds					
							FMA score				Affected side			Unaffected side		
							1th	2nd	3rd	4th	1th	2nd	3rd	1th	2nd	3rd
sham	7	51	M	44	L	R	32	33	42	45	80	93	90	52	61	61
sham	9	39	M	25	R	R	32	36	42	39	—	—	—	41	42	43
sham	11	59	M	52	L	R	33	41	46	57	91	89	84	46	39	42
sham	31	47	M	10	R	R	35	—	40	40	—	—	—	45	44	39
sham	32	67	M	52	L	R	42	43	45	46	60	75	65	36	44	37
sham	18	70	F	19	L	R	23	23	25	26	—	—	—	38	44	36
sham	19	59	M	44	L	R	25	29	24	28	—	—	—	48	55	64
sham	21	58	M	29	R	L	24	28	32	37	—	—	—	62	62	54
sham	30	58	M	25	L	R	19	20	22	24	—	90	90	46	54	58
tDCS	1	29	M	12	L	L	41	51	50	51	76	64	68	47	45	41
tDCS	5	54	M	28	R	R	38	29	34	42	86	78	57	33	32	37
tDCS	15	48	F	49	R	R	42	39	42	46	—	—	—	44	46	49
tDCS	27	65	M	27	R	R	42	41	45	48	—	—	—	45	37	37
tDCS	29	57	F	10	R	R	41	40	40	44	—	—	—	68	66	65
tDCS	6	38	F	29	L	R	28	38	41	42	—	—	—	38	32	34
tDCS	10	60	F	51	L	R	21	26	22	31	80	74	68	60	58	58
tDCS	25	59	M	13	R	R	23	31	28	31	79	—	72	54	—	58
tDCS	37	65	M	86	L	R	26	28	29	28	—	—	71	59	60	59

Note: *Subject #31 was not available for the 2^nd^ FMA measurement. **Subject #25 did not get 2^nd^ RMT measurement due to technical difficulty. All other ‘−’ labels in the RMT reading denote undetectable motor evoked potential.

### Lesion volume and location

Figure 2 shows the lesion maps of the sham group, tDCS group, and all patients combined. Lesion mostly occurred in the middle section of the cortical spinal tracts. The volume of the lesion was not significantly different between the tDCS (3737.9 ± 4048.0 mm^3^) and the sham (2750.1 ± 4373.4 mm^3^; p = 0.63) group.

### DTI results

Compared to healthy controls, stroke subjects showed significantly lower FA at baseline along the ipsilesional corticospinal tract (*cst*), starting from the cerebral peduncle up till the motor cortex. Lower FA was also seen in bilateral *cc*, spanning from the first to the fifth sections (Fig. 3a). There was no significant difference in the baseline FA between the tDCS and sham groups. After training, the tDCS group showed higher FA in the 2nd section of the ipsilesional *cc*, and the 3rd, 4th, and 5th sections of *cc* bilaterally (Fig. 3c) while the sham group showed decreased FA in the 2nd section of the ipsilesional *cc* (Fig. 3d) compared to the baseline. Compared to the sham group, FA change was higher in the tDCS group in the ipsilesional *cst* and *cc*, as well as anterior to middle *cc* of the contralesional side (Fig. 3b). Further ROI analysis on the sections of *cc* showed that in the ipsilesional *cc* where the tDCS group had increased FA (ROI1–4 in Fig. 3c), the mean Dr all decreased significantly (p = 0.0005, 0.046, 0.00263, and 0.0019, respectively) while only ROI3 and 4 showed Dp increase (p = 0.013 and 0.045, respectively). In the contralesional *cc* (ROI5–6 in Fig. 3d), Dr decreased (p = 0.03 and 0.001, respectively) but not Dp. In the single region where the sham group showed FA reduction in the premotor and supplementary motor region of the ipsilesional *cc*, no significant change in Dp or Dr was detected (Fig. 3e).

### CBF results

**Figure 4:**
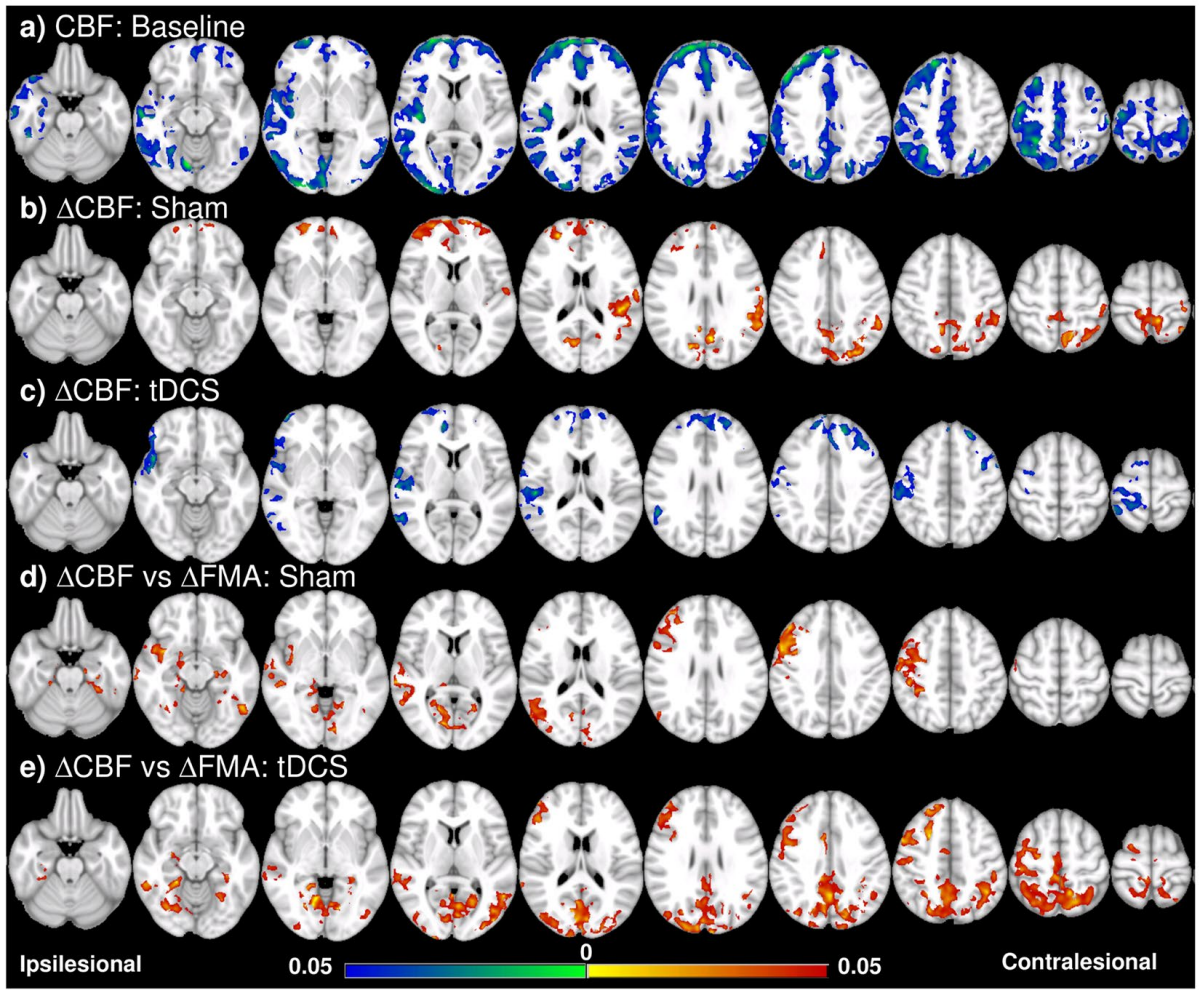
Voxel-wise CBF analysis and behavioral correlation. (**a**) CBF of all the patients (tDCS and sham groups together) is lower especially in the ipsilesional hemisphere compared to the control at the baseline. (**b**) CBF increase in the frontal and contralesional side after training in the sham group. (**c**) CBF decrease in the frontal and ipsilesional side after training in the tDCS group. (**d**) ∆CBF positively correlates with ∆FMA in the sham group in the ipsilesional side, including the pre/postcentral cortices, angular gyrus, lateral occipital cortex, and middle temporal gyrus, and the ventral occipital lobes in both sides; (**e**) in the tDCS group, positive correlation between ∆CBF and ∆FMA is in similar regions as well as the posterior and superior part of the two hemispheres including the posterior parietal cortices. Colorbar represents p-value with hot color as increase (or positive correlation) and cold color as decrease (or negative correlation) (p < 0.05, FWE corrected).

Voxel-wise comparison showed consistent CBF measured in the healthy control (data not shown) and widespread reduction of the baseline CBF in the stroke subjects compared to the healthy control. The affected regions include bilateral frontal and occipital lobes, ipsilesional temporal and parietal lobes, and part of the pre/post central gyri on the contralesional side (Fig. 4a). No baseline CBF difference was found between the tDCS and the sham groups. After training, CBF was increased bilaterally in the frontal poles, paracingulate/cingulate gyri and precuneous/cuneous cortices, and contralesionally in central/parietal opercular cortex and supramarginal gyrus in the sham group (Fig. 4b). On the contrary, CBF was decreased in the tDCS group in the bilateral frontal poles, and ipsilesional paracingulate/cingulate gyri and postcentral cortices (Fig. 4c).

**Figure 5:**
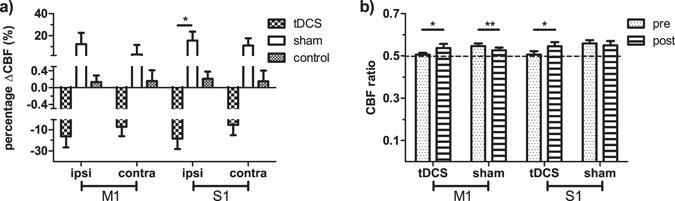
ROI analysis on CBF. (**a**) CBF in the primary motor (M1) and somatosensory (S1) cortices tends to be reduced after tDCS while increased after sham. Ipsi: ipsilesional side; contra: contralesional side. (**b**) The CBF asymmetry ratios in the primary motor and somatosensory cortices were increased after tDCS while decreased in the primary motor cortex in the sham group. For the control group, the change of CBF is the difference between the 2 scans. *p < 0.05; **p < 0.01. Error bar represents SEM.

ROI analysis further showed that CBF in both ipsilesional and contralesional M1 were increased after MI-BCI training while decreased following MI-BCI-tDCS particularly in the ipsilesional side, resulting in reduced and increased CBF asymmetry ratio in the sham and tDCS groups respectively (Fig. 5a,b). Similar trends were also found in S1. The CBF change after intervention in the patient groups was much larger than that seen in the control group, and significantly different between the sham and tDCS groups in the ipsilesional somatosensory cortex (p = 0.0186).

### Behavioral correlates

Positive correlation between the CBF change and the FMA change was found in both groups (Fig. 4d,e). For the sham group, the correlation was mostly seen in the ipsilesional side, including the pre/postcentral cortices, angular gyrus, lateral occipital cortex, and middle temporal gyrus. Positive correlation also showed in the ventral occipital lobes in both sides including the intracalcarine cortex and lingual gyrus. For the tDCS group, positive correlation was found in similar regions as well as the posterior and superior part of the two hemispheres including the posterior parietal cortices. It should be noted that these positive areas are not detected in Fig. 4c and hence represent sub-threshold changes. In the tDCS group, the posterior and superior parts of the brain showing correlation correspond to the areas interconnected by posterior transcallosal fiber bundles where increased FA was seen (ROI4&6 in Fig. 3c). We found no correlation between the baseline FA and baseline FMA, between the change of FA and the change of FMA, or between the baseline FA and the change of FMA, indicating that the FA was not related to the motor function measured by FMA. However, the change of Dp in the ROI4 (Fig. 3c) was positively correlated with the change of FMA (r = 0.72, p = 0.03).

### RMT results

**Figure 6:**
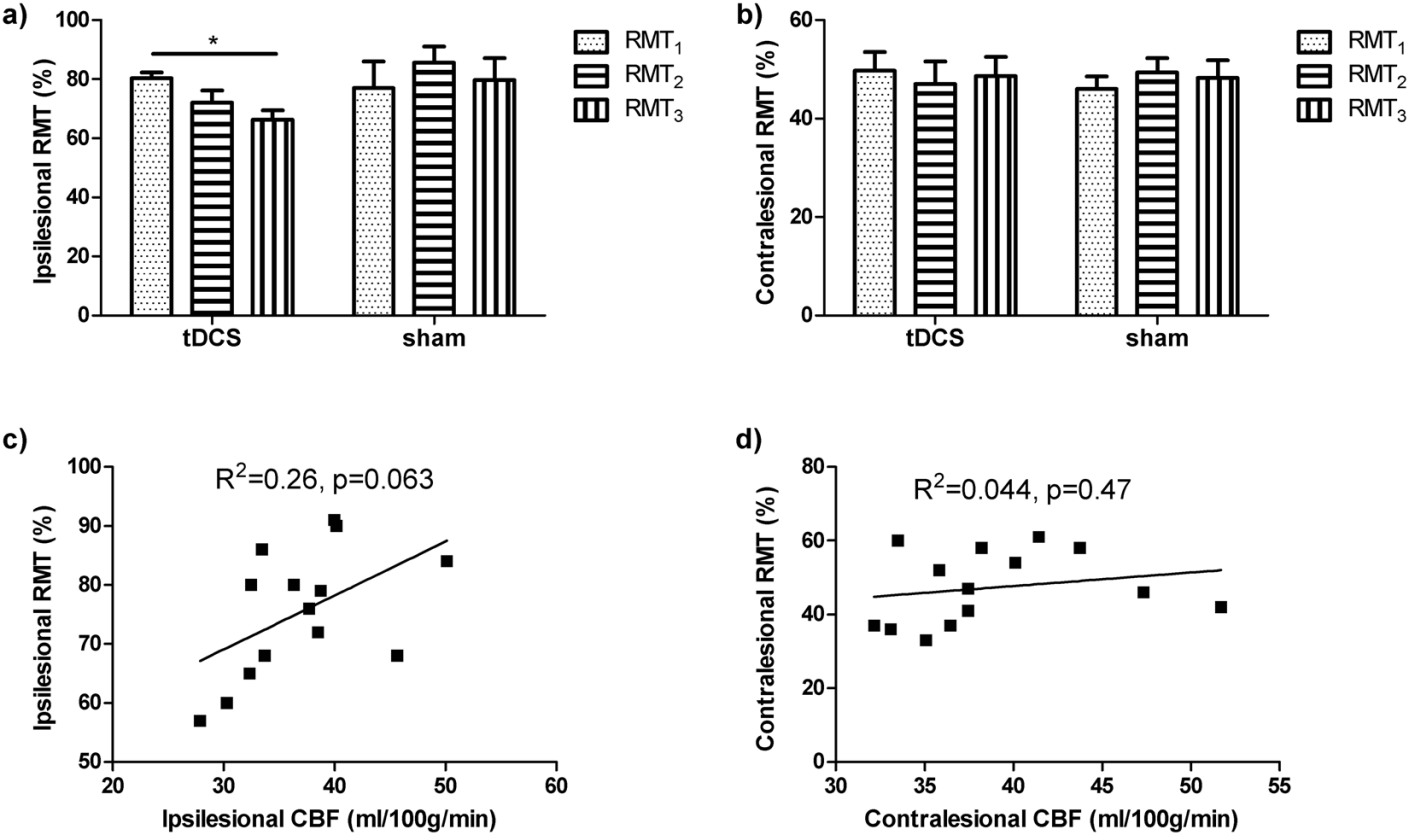
Training effects on RMT and its relationship with CBF. (**a**) Ipsilesional and (**b**) contralesional RMT before (RMT1) and after (RMT2 and RMT3) training. Ipsilesional RMT were decreased in the tDCS, while no change in the contralesional side, nor in the sham group (*p < 0.05, error bar represents SEM). CBF of the primary motor cortex in all the patients with measurable RMT shows trend of correlation with RMT in the (**c**) ipsilesional but not (**d**) contralesional side.

MEPs in the affected arm were not detectable in 5 out of 9 subjects in the tDCS group and 6 out of 9 subjects in the sham group. Therefore results for RMT were only available in 7 stroke subjects. Ipsilesional RMT was found to decrease after MI-BCI training in tDCS but not sham (Fig. 6a). The ipsilesional RMT showed a trend of positive correlation with the CBF in M1 (Fig. 6c). No significant change in RMT measures and correlation with CBF was found on the contralesional M1 for either tDCS or sham (Fig. 6b and d). Furthermore, paired-pulse TMS measures of SICI and ICF changes showed no significant differences between groups (tDCS vs. sham) and hemispheres (contralesional vs ipsilesional M1) at the two post-training time points. It should be noted, however, that a trend to significance was observed in the SICI changes in the contralesional M1 of the tDCS group at the first (p = 0.067) and second (p = 0.062) post-training time points.

## Discussion

Previous studies have demonstrated positive effects on motor recovery by using either MI-BCI^1, 4^ or tDCS^12, 13^ in stroke patients. Our preliminary study also demonstrated improvements in motor performance, though no additional enhancement by combining tDCS with MI-BCI. Interestingly, neuroimaging showed significant changes in WM integrity and CBF by tDCS even at four weeks after the rehabilitation training. FA measure of WM integrity was increased in the ipsilesional *cst* and the *cc* connecting the bilateral motor, somatosensory and parietal cortices in the tDCS group. The tDCS also led to a long-lasting decrease of CBF and RMT (i.e., increase of cortical excitability) in the ipsilesional sensorimotor cortex. The CBF change in the bilateral sensorimotor, parietal and occipital cortices also positively correlated with the behavioral change, consistent with the increase of FA in the *cc* that connecting them. The very different neuroplasticity induced by tDCS compared to MI-BCI alone indicates that different mechanisms were facilitated although the difference was not well reflected in the behavior readout used.

The corticospinal tract, projecting directly from the primary motor cortex to the spinal cord, is one of the most critical WM tracts for normal motor functions. The structural integrity of the ipsilesional *cst* has been associated with the motor function of the affected arm/hand in stroke patients^36–42^. In this study, larger FA increase in the ipsilesional *cst* was seen in the tDCS group, suggesting that combining tDCS with MI-BCI facilitated recovery of the ipsilesional *cst*. Previous studies suggested that the increase of FA during recovery could be associated with axonal regeneration, remyelination, gliosis, or any combinations of these events^43–45^. By inspecting the diffusivities, we found that the increase of FA was mainly due to decreased Dr, and partially due to increased Dp in certain regions, implicating the occurrence of remyelination and axonal regeneration. Studies have reported that the extent of damage in the ipsilesional *cst* could predict the motor recovery^21, 22, 46–49^, and the extent of FA increase correlates with the extent of motor recovery^50^. The correlation was not seen in our results, possibly due to the relatively long time post stroke at the study enrollment (range 10–86 months, median 29 months), and the wide range of age (29–70 years) and baseline motor performance (pre-training FMA ranging from 19.5 to 46), the short intervention duration, or lack of motor execution training on top of the motor imagery training.

Another WM tract that plays important roles in normal motor execution is the corpus callosum, which connects the left and right cortical areas including the motor and somatosensory cortices. We found that the baseline FA in the ipsilesional part of *cc* was affected by the stroke even though it is remote from the lesion. MI-BCI training led to significant FA reduction in the ipsilesional *cc* in the section subserving the premotor and supplementary motor areas. On the other hand, combining with tDCS improved the structural integrity of the middle and posterior parts of the *cc*, which connects the motor and sensory cortices. Previous studies have suggested that the interhemispheric inhibition is critical in the normal unilateral or bilateral movements^51, 52^, which can be interrupted by the impairment of *cc* ^51, 53^. A recent DTI study in chronic stroke patients found that reduced FA in the transcallosal M1-M1connection was negatively associated with motor recovery following tDCS and physical/occupational therapy, but not related to baseline motor function^21^. Furthermore, they found the association between white matter microstructural integrity and motor recovery was stronger in the *cc* than in the *cst*. In another study, lower FA in chronic stroke patients compared with healthy control was found in the transcallosal S1-S1 rather than M1-M1 connection^54^. Consistent with these finding, we found increase of FA in the tDCS group more in the sensory (bilaterally) than the motor (only on the ipsilesional side) section of *cc*, suggesting an important involvement of the sensory system in motor function.

The preliminary findings of widespread CBF reduction in stroke patients and the positive correlation between CBF change and FMA change in both sham and tDCS groups are in line with other studies where restoration of perfusion in hypoperfused areas is related to enhanced clinical outcomes^27, 55, 56^. Interestingly, the CBF was reduced in the tDCS group especially in the ipsilesional sensorimotor area. So far, only few studies have reported the short-term effect of tDCS on CBF. A positron emission tomography (PET) study of the CBF in healthy subjects reported that anodal tDCS predominantly increased CBF in many cortical and subcortical areas while cathodal tDCS decreased CBF^57^. A study using ASL concurrently with tDCS found that CBF increased by either anodal or cathodal tDCS during stimulation, and then continued to be increased a few minutes after anodal stimulation while decreased after cathodal stimulation^58^. Another ASL study of tDCS in healthy volunteers showed widespread CBF decrease within 10 minutes after anodal or cathodal tDCS compared to the period during stimulation^59^. This is consistent with our observation of bilateral CBF reduction in the sensorimotor cortices. Although the ipsilesional CBF correlated with the RMT, the overall mechanisms that lead to such a long-lasting down-regulation of CBF and its implication on the motor function need further investigation.

Another interesting finding in the tDCS group is that the regions where the increase of CBF correlated with functional improvement corresponded well with the cortices subserved by the *cc* sections with higher FA. Earlier DTI and fMRI study showed that greater damage to the *cc* was associated with stronger activation in bilateral motor areas and poorer motor outcome^60^. In accordance with this finding, our results suggested that improved transcallosal connectivity associates with recruitment of other functional cortices and/or functional integration, which facilitates the motor recovery. This is different from using MI-BCI alone in the sham group, where the functional recovery is correlated with unilateral, not bilateral, CBF change.

ROI analysis of the CBF in the primary motor and sensory cortices showed that ipsilesional CBF was lower than the contralesional side. After MI-BCI training, CBF was increased on both sides but more on the ipsilesional side, indicating a re-balance process during recovery. This finding supported the hypothesis that balanced interhemispheric interaction is critical for normal motor function^53, 61^. On the other hand, tDCS enlarged the interhemispheric difference by reducing the CBF on the ipsilesional more than the contralesional side. Interestingly, the enlarged CBF imbalance did not lead to lower function improvement compared to the sham group, possibly because of the positive effects of tDCS on strengthening the interhemispheric connectivity of the *cc*. Cumulating evidence from magnetoencephalography, electromyography, TMS, and fMRI studies have suggested that the contralesional motor cortices may play either a supporting^62–65^ or hindering^53, 66–68^ role in the functional recovery of the affected limb, depending on factors such as time post-stroke, lesion size and location^69–71^. The exact mechanisms of the interhemispheric interaction and how these may influence post-stroke functional recovery remain to be investigated.

Either enhancing ipsilesional M1 excitability by anodal tDCS, or inhibiting contralesional M1 by cathodal stimulation, has been demonstrated to improve motor function in stroke patients^13, 14, 72, 73^. By combining the two approaches, bi-hemispheric tDCS has been demonstrated to be about 50% more effective than uni-hemispheric tDCS in promoting motor function in healthy subjects^74^. A recent study in stroke patients further showed that 5 sessions of physical motor training combined with bi-hemispheric tDCS produced larger functional improvement than physical therapy alone, and the after effects outlasted the intervention by at least 1 week^75^. Our bi-hemispheric tDCS intervention, despite with more training sessions, did not show behavioral advantage over MI-BCI alone. Possible reasons include weaker current intensity applied (1 vs 1.5 mA), shorter stimulation duration (20 vs. 30 min), later assessment of motor function post training (3 vs 1 week) in our study. On the other hand, studies have suggested that activity in contralesional motor cortices may interfere with the motor recovery of the affected limb in some patients^53, 66–68^. In particular, decreased contralesional motor excitability may mitigate functional recovery^76^. In our study, we didn’t find changes in contralesional motor excitability and hence it may not be the reason.

There are several limitations in the current study. First of all, the sample size is relatively small, especially in the CBF analysis with some data removed due to poor image quality. The sample size for RMT measurement was even smaller because no RMT can be determined in several patients even at the maximum stimulation intensity. This has been an issue for most BCI or tDCS studies in stroke rehabilitation. Multi-center study will be needed to not only increase the sample size but also to evaluate the reproducibility. Secondly, though exclusion criteria was carefully set aiming to exclude other neurophysiological or psychiatric confounding conditions, considerable pathological heterogeneity still exists in the patient cohort in terms of the post-stroke time, the size and location of the lesion, and the baseline motor function. All these factors, together with demographic factors such as age, sex, whether the preferred hand was affected, and life-style may contribute to the observed variance in the structure, function and behavior. In our pilot analysis incorporating these factors (except for life-style which was not available) as co-variants into the statistical models relating DTI metrics with behavior score, no significant difference in the results was observed (data not shown). A more homogeneous sample pool will certainly boost the statistical power, though difficult in practice. Thirdly, the impaired vascular function and potentially lower baseline CBF in some patients may affect the accuracy for determination of functional improvement using hemodynamic-based measures, including CBF and blood oxygenation level dependent contrast. In our analysis, percent CBF change was used to minimize the bias of the baseline CBF. In the ROI analysis, no deficiency in CBF was observed in the sensorimotor cortex and hence the analysis on absolute CBF in this area would still represent tissue function. Fourthly, the montage of tDCS used (anode on the M1 and cathode on the contralateral supraorbital area) would make the current passing through not only the primary motor but the premotor and S1 areas as well. Therefore the involvement of these other areas in the plasticity can not be ruled out. Finally, image flipping was used in our processing pipeline to facilitate voxel-wise analysis. The brain is not symmetric along the midline in terms of both structures and functions^77^. Therefore image flipping may introduce error in the image registration and variance into the statistical analysis.

## Conclusion

In this preliminary study, we found the enhanced neuroplasticity in WM structure and cortical function by tDCS following a combined MI-BCI and tDCS training in chronic stroke patients. Although no further behavioral improvement was found, the long-lasting increase of ipsilesional motor excitability and WM integrity between motor-related areas indicate the facilitating effect of tDCS. Our results support the critical role of interhemispheric interaction in the motor functional recovery. The knowledge of the neuroplasticity in terms of structural and functional alterations and adaptations during cortical neuromodulation and motor learning would facilitate future research to design more effective rehabilitation strategy for chronic stroke patients.

## Electronic supplementary material

Supplementary Figure
