# Supplementary material for: Brain plasticity following MI-BCI training combined with tDCS in a randomized trial in chronic subcortical stroke subjects: a preliminary study
Source: Sci Rep. 2017 Aug 23;7:9222. doi: 10.1038/s41598-017-08928-5 (PMC5569072; doi:10.1038/s41598-017-08928-5)
Supplement: Supplementary file 1 — Supplementary Figure [file 41598_2017_8928_MOESM1_ESM.doc]

**Brain plasticity following MI-BCI training combined with tDCS in a randomized trial in chronic subcortical stroke subjects: a preliminary study**

Xin Hong, Zhong Kang Lu, Irvin Teh, Fatima Ali Nasrallah, Wei Peng Teo, Kai Keng Ang, Kok Soon Phua, Cuntai Guan, Effie Chew, and Kai-Hsiang Chuang

**Supplementary Figure.**


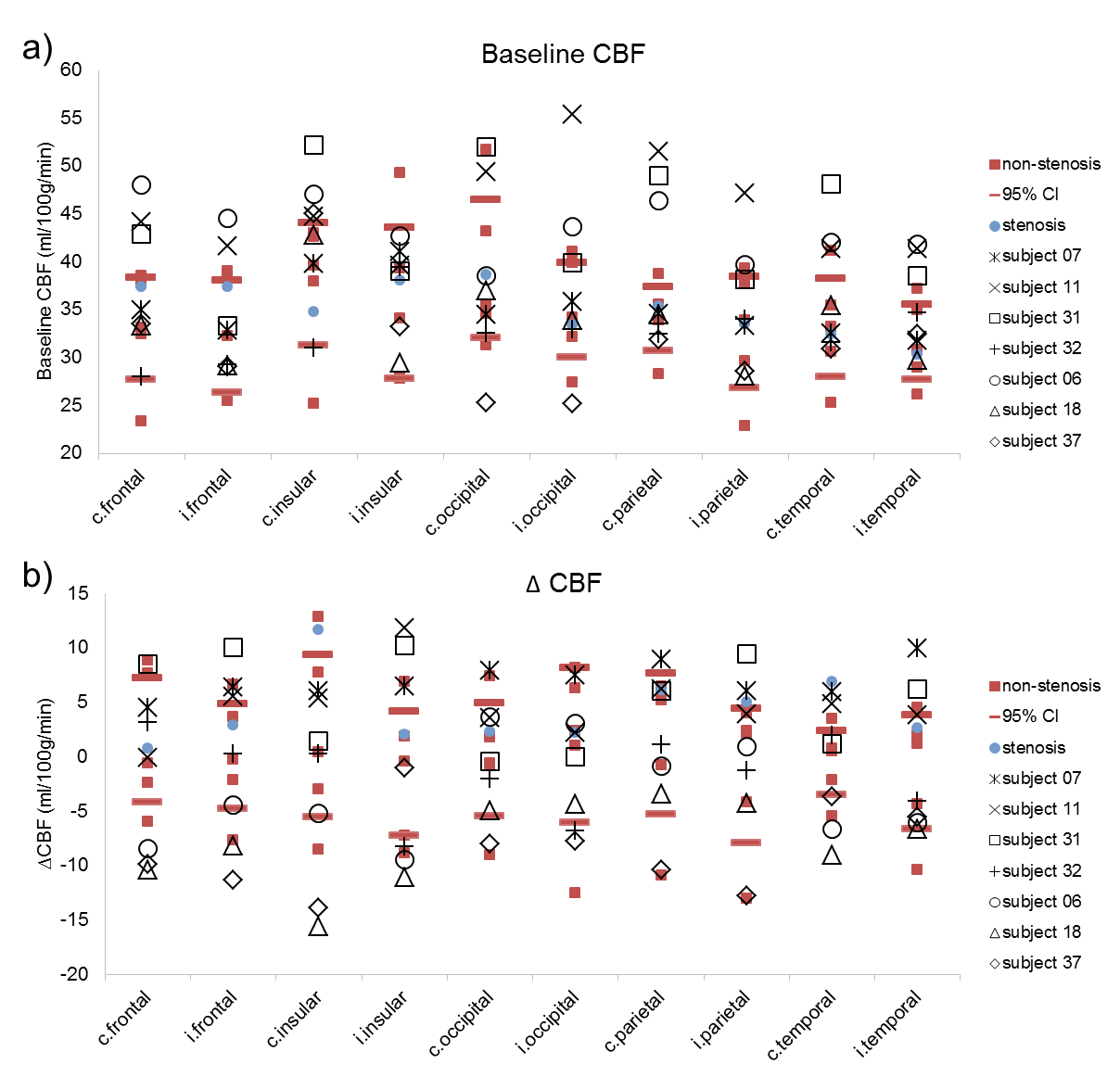


Figure S1. CBF and its change after intervention in patients with or without stenosis. The baseline CBF (a) and CBF change after intervention (b) in the contra-lesional (labelled as c.) and ipsi-lesional (labelled as i.) lobes of the non-stenosis (n=5; red square), stenosis (n=1; blue dot) and unconfirmed (n=7; black symbols) patients. The short bars represent the 95% confidence interval (CI) of mean of the non-stenosis patients.
